# Where Do MAIT Cells Fit in the Family of Unconventional T Cells?

**DOI:** 10.1371/journal.pbio.1000070

**Published:** 2009-03-31

**Authors:** Laurent Gapin

## Abstract

Mucosal-associated invariant T cells are newly identified subpopulation of T cells. A new study highlights their developmental pathway and functional features that allow these cells to assume a unique position in the family of unconventional T cells.

The immune system protects the body by relying on specialized cells that can recognize billions of pathogenic foreign particles. This recognition is achieved through the generation of a highly diverse collection of antigen receptors, expressed at the surface of B and T lymphocytes, that are made by random recombination of V, (D), and J segments and trimming/addition of nucleotides at the junctions between these rearranged gene segments. Such mechanisms ensure the creation of a vast repertoire of receptors that is necessary to survey the multitude of antigens that continuously challenge the immune system. In contrast to the enormous receptor diversity of antigen receptors found on conventional B and T cells, several lymphocyte subpopulations with limited repertoire diversity have been identified. This includes the B1 B cell subset [1], some γδ T cell subpopulations [2], the natural killer T (NKT) cells [3], and the mucosal-associated invariant T (MAIT) cells [4]. These discrete subpopulations tend to be phylogenetically conserved, localize to specific tissues, and are often considered to act at the frontier between innate and adaptive immunity: like adaptive immune cells, they generate antigen receptors through V(D)J recombination, but like innate immune cells, they tend to recognize a limited set of antigens and respond quickly to an immune challenge.

## Invariant NKT Cells

The majority of NKT cells express a T cell receptor (TCR) that is the product of a canonical Vα14-Jα18 (Vα24 in humans) rearrangement, with the junctional region invariant at the amino acid level. This Vα14 invariant chain is co-expressed with a limited set of Vß chains, which together form a functional αß receptor. In mice and humans, this NKT cell population is referred to as type I NKT cells, or invariant (*i* NKT) cells [5]. While the majority of “conventional” αß TCR^+^ cells respond to peptides bound to conventional major histocompatibility complex class I (MHCI) and class II (MHCII) molecules, *i*NKT cells recognize glycolipid antigens presented by the MHCIb molecule, CD1d [6]. A hallmark feature of MHCI and MHCII proteins is the extensive polymorphism in residues that constitute their peptide-binding groove, which forms the basis for differential peptide binding, thymic repertoire formation, and allograft rejection. In contrast, CD1d molecules are essentially nonpolymorphic among inbred strains of mice and humans, suggesting that these molecules likely evolved to present only a limited set of glycolipidic antigens. The high degree of conservation of the canonical TCR and CD1d molecules allows for interspecies cross-reactivity, with mouse *i* NKT cells recognizing human CD1d and vice versa, indicating the likely importance of these cells in the immune system.

## MAIT Cells

Like *i* NKT cells, MAIT cells are also characterized by the expression of a semi-invariant TCR rearrangement (Vα7.2-Jα33 in humans and Vα19-Jα33 in mice), with a junction of constant length but some variability in two junctional codons [7,8]. This invariant α chain is also found preferentially associated with a limited number of TCRß chains (Vß2 and Vß13 chains in humans and Vß6 and Vß8 chains in mice). Such TCRs recognize another MHCIb molecule highly conserved in mammals, MHC-related molecule 1 or MR1 [9]. Compelling evidence that MR1 molecules likely present antigens to MAIT cells has been published [10]; however, the nature of these antigens remains currently unknown. This expression of a highly conserved invariant Va chain coupled to the recognition of a non-polymorphic MHCI-like molecule, their preferential homing to specific tissues, and their currently defined phenotype (see below) has won this population the acronym of mucosal NKT or *m*NKT cells [11].

Developing new reagents has helped us define invariant T cell populations. For example, the generation of CD1d tetramers loaded with the prototypical glycolipid antigen, α-galactosylceramide, permitted the specific detection of *i*NKT cells in both mice and humans [12]. Using this reagent, important progress in our understanding of the ontogeny, developmental requirements, phenotype, tissue localization, and function(s) of *i* NKT cells has been achieved [13,14]. Unfortunately, until recently the only way to detect MAIT cells in humans and unmanipulated mice was through molecular techniques targeting the invariant TCRα chain [9], which prevented the in-depth exploration of MAIT cell biology.

**Figure 1:**
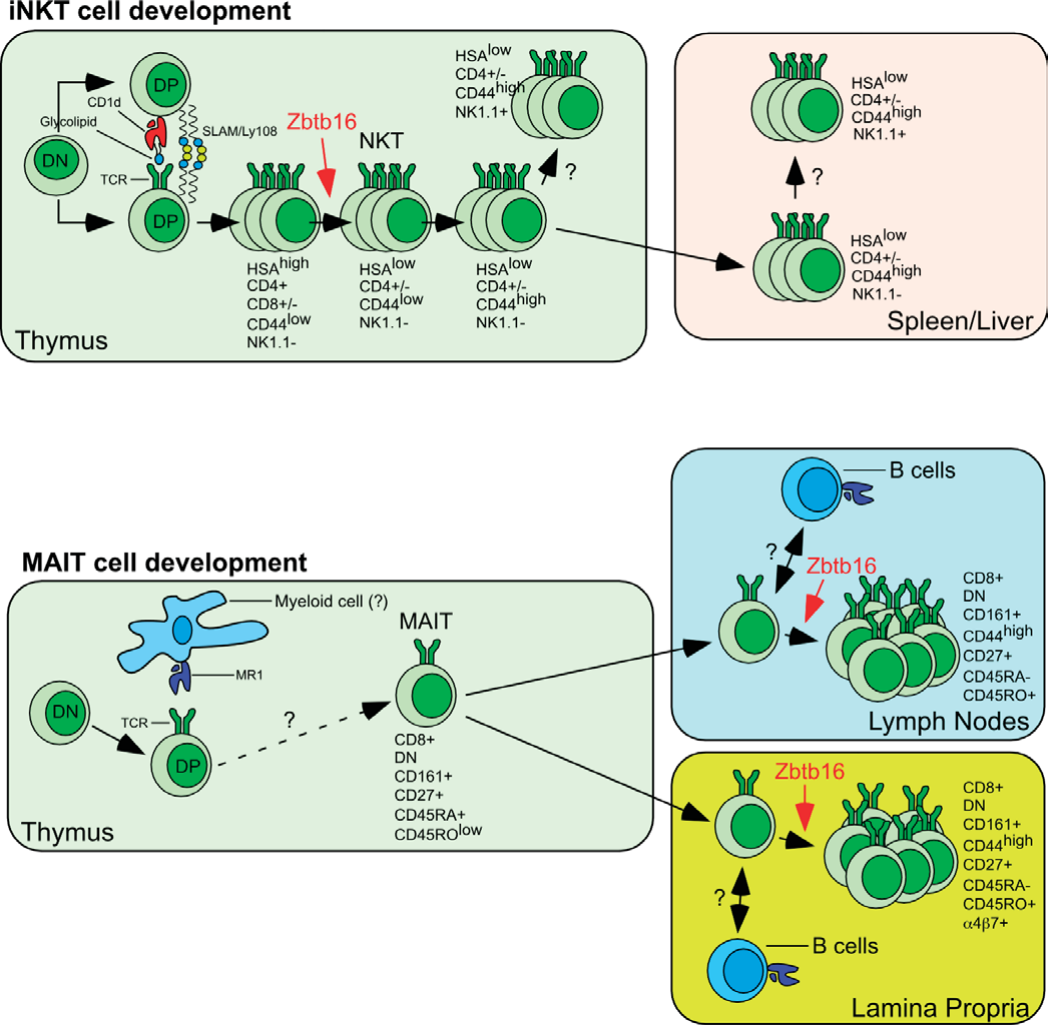
Schematic View of *i*NKT Cell and MAIT Cell Development NKT and MAIT cells both arise in the thymus from uncommitted DN precursors. The cells progress to the DP CD4^+^ CD8^+^ stage, where they presumably randomly rearrange the TCR. Thymocytes that express a TCR that interacts with CD1d bound to self-glycolipid, expressed by other DP thymocytes, enter the *i* NKT lineage. Homotypic interactions between SLAM and Ly108 molecules, also expressed at the surface of DP thymocytes, are required for progression into the *i* NKT cell lineage. Thymocytes that express a TCR that interacts with MR1 expressed at the surface of an undefined myeloid cell type are positively selected into the MAIT cell lineage. Such lineage commitment does not require SLAM/SAP signaling. Whether other specific signaling pathways are required for the generation of MAIT cells remains to be determined. After selection, *i*NKT cell precursors undergo a series of differentiation steps characterized by the sequential expression of several cell surface markers (HSA [heat-stable antigen], CD44, and NK1.1), as depicted. How MAIT cells mature to their characteristic phenotype is currently unknown. The earliest *i*NKT cell precursor found in the thymus expresses the highest level of the transcription factor Zbtb16. Zbtb16 expression level then decreases as the cell progresses further in the differentiation pathway. At least four distinct immature *i* NKT cell populations have been identified in the thymus. Most *i*NKT cells that emigrate from the thymus do so in an immature form (CD44^high^, NK1.1^−^) and can continue their final maturation in the periphery (spleen/liver). Some mature *i*NKT cells also migrate to the periphery, but many remain as long-term residents of the thymus. MAIT cells leave the thymus with a naïve phenotype and expand in peripheral organs such as the lymph nodes and the mucosa epithelia (such as the lamina propria), where they expand upon interactions with B cells. This cell expansion is associated with the expression of Zbtb16 and the acquisition of a “memory” phenotype.

In this issue of *PLoS Biology,* Emmanuel Martin and colleagues at the Lantz laboratory now report the isolation of a monoclonal antibody directed against the human Vα7.2 chain that is part of the MAIT TCR [15]. Just as the generation of the CD1d tetramer revolutionized how investigators could study *i* NKT cells, one might predict that the development of this monoclonal antibody will profoundly impact the MAIT cell field. Using this new reagent, as well as several genetically modified mice, the Lantz laboratory has focused their first study on the development of MAIT cells. The status quo of our understanding of MAIT cell development is depicted in Figure 1 and compared to *i* NKT cell development.

Using molecular techniques to measure the amount of invariant TCRα chain in peripheral organs, it was previously established that MAIT cell development is dependent upon the expression of MR1 on bone marrow-derived cells. It was also shown that MAIT cells accumulate in the epithelial layer of the intestinal lining in a process dependent on commensal flora and on the presence of B cells, suggesting the possibility that MAIT might in fact be directed against some microbial antigens presented by MR1 molecules at the surface of B cells [9]. However, it is known that some intraepithelial T lymphocytes might be generated by extra-thymic lymphopoiesis, and given that MAIT cells could not be detected in the thymus of either humans or wild-type mice, their origin and developmental requirements remained elusive. In this new study, the authors were able to detect MAIT cells in the human thymus and in mouse fetal thymic organ cultures. Together with the observation that MAIT cells are absent in athymic *nu/nu* (nude) mice [8], these results make a compelling case that MAIT cells, like *i* NKT cells, likely develop in the thymus. In agreement with previous findings, MAIT cells were essentially found within the CD4^−^ CD8^−^ double negative (DN) and CD8αß^+^ populations, with very few CD4^+^ cells expressing the canonical TCR. In addition, some cells appear to express the homodimer CD8α α in humans. Interestingly, human cord blood MAIT cells, as well as mouse MAIT cells, displayed a naïve phenotype (i.e., lacked expression of receptors associated with prior antigen exposure) with no significant expression of several markers (including NK1.1, CD25, CD69, and ICOS) that had been previously used to identify MAIT cells in other TCR transgenic mice [16,17]. The reasons for such a discrepancy remain unclear at the moment but might be related to differences in experimental procedures and/or mouse colony housing. By contrast, in human adult blood, MAIT cells, like *i*NKT cells, have a memory phenotype (i.e., expressed several markers usually found on antigen-experienced T cells) and expressed the NK marker CD161 (NKRP1A), which, importantly, is not the human ortholog of the mouse NK1.1 marker (NKR-P1B/C in B6 mice) [18].

What do all these results mean? The simplest and most logical explanation is that MAIT cells originate and undergo positive selection in the thymus and are exported to peripheral organs while retaining their naïve phenotype. Because B cells are absent from fetal thymic organ cultures, the results also suggest that B cells are not required for the development of MAIT cells per se but might be needed for the expansion and/or final maturation of MAIT cells in the periphery. In support of this hypothesis, Martin et al. performed experiments adding back B cells, either expressing or lacking the MR1 molecule, to transgenic mice engineered to express only the MAIT TCR at the surface of all T cells. Originally, very few MAIT cells were detected in the peripheral organs of these animals, but their number increased over 10-fold during a two-week period after B cells were transferred into the mice. Interestingly, MR1-deficient B cells were almost as efficient at inducing this expansion as MR1-expressing cells, suggesting that cognate interaction between the invariant TCR and MR1 might not be necessary for this expansion. Such results might perhaps have been expected in light of the extremely low to undetectable levels of MR1 expression at the surface of ex vivo purified cells [19]. Indeed, although MR1 mRNA expression seems ubiquitous [20,21], endogenous cell surface expression of MR1 in vivo remains unclear. Further work will be necessary to decipher the exact role of B cells in MAIT cell expansion and to determine which MR1-expressing cell type is responsible for the positive selection of MAIT cells in the thymus.

## MAIT Cells Are Not *i* NKT Cells

*i*NKT cells also develop in the thymus and are positively selected by bone marrow-derived double positive (DP) thymocytes that express CD1d. Following positive selection at the DP stage, *i* NKT cells also undergo an orchestrated maturation process that ultimately leads to the acquisition of their activated NK-like phenotype. However, when *i* NKT cells leave the thymus they are still in an immature stage (as defined by the absence of expression of NK receptors such as NK1.1) and fulfill their terminal maturation in the periphery [14]. As for MAIT cells, it is currently unclear what signal(s) regulate *i* NKT cell terminal maturation. The adaptor SLAM-associated protein (SAP) and the transcription factor Zbtb16 (PLZF) were both recently reported to be essential to the proper development of *i* NKT cells [22–24]. Martin et al. examined whether these two molecules also influence the development of MAIT cells. While no *i*NKT cells could be detected in the blood of five SAP-deficient patients, MAIT cells were present with a normal frequency and phenotype. These results suggest that, in contrast to *i* NKT cells, the cooperative engagement of homophilic receptors of the signaling lymphocytic-activation molecule (SLAM) family, whose signaling properties are mediated through SAP, is not involved in the development of MAIT cells. The results for Zbtb16 turned out to be more complicated. Zbtb16 is not expressed in mouse MAIT cells, which, in this study, have a naïve phenotype, but is found at high levels in human blood MAIT cells that express an activated/memory phenotype. The correlation between the “memory” phenotype of MAIT cells and the expression of Zbtb16 clearly associates this transcription factor with the acquisition of this particular phenotype. In fact, recent work showed that forced expression of Zbtb16 in T cells, using transgenesis, induced T cells with an antigen-experienced phenotype that could quickly produce the cytokine interferon-γ upon stimulation, a characteristic of innate-like immune cells [25]. The questions remain as to what signals induce Zbtb16 expression in *i*NKT cells and human MAIT cells and what species-specific differences between mice and humans are responsible for this differential MAIT cell phenotype.

## MAIT Cells Continue To Intrigue Us

While certain similarities indeed exist between the two *i* NKT and MAIT cell populations, the current study clearly outlines major differences in their developmental requirements and phenotype. Furthermore, these new findings prompt us to re-evaluate many important questions regarding MAIT cells. First, what are the effector functions of MAIT cells? In agreement with their previously defined effector/memory phenotype and similarly to *i* NKT cells, MAIT cells had previously been shown to rapidly produce effector cytokines (including interleukin-4, interleukin-5, interleukin-10, tumor necrosis factor-α, and interferon-γ after activation via MR1 [16,17]. However, Martin et al. now challenge previous reports indicating that MAIT cells have an activated phenotype in mice. This inconsistency makes it particularly important to determine whether MAIT cells derived from the current transgenic mice and human Vα7.2^+^ CD161^+^ cells indeed behave like innate lymphocytes by producing rapidly effector cytokines upon stimulation, or not.

Second, where are MAIT cells located? MAIT cells derive their name from the findings that the invariant TCRa chain is enriched (in humans and mice) in lymphocyte preparations derived from the gut (the lining lamina propria and Peyer's patches) and perhaps the lungs [9]. The current study did not assess whether MAIT cells might be located in other tissues, but the newly made anti-Vα7.2 monoclonal antibody will certainly facilitate this task in the future. Strikingly, Martin et al. showed that MAIT cells represent up to 1%–4% of total blood T cells, which greatly exceeds the reported frequency of *i*NKT cells (generally less than 0.2% of total peripheral blood lymphocytes [26]). The high frequency of MAIT cells could potentially correlate with important functions in humans. The next challenge will be to determine what these functions might be and whether the frequency and number of MAIT cells varies under different conditions, such as during infections or in the presence of tumors or autoimmunity. Due to their prominence in the intestine, it was previously suggested that MAIT cells might have a positive role in regulating the secretion of immunoglobulin A molecules that are resistant to enzyme degradation in the harsh environment of the digestive tract [9,27]. This possibility awaits experimental confirmation. Other reports have also suggested the accumulation of MAIT cells in central nervous system lesions from multiple sclerosis autopsy samples [28] and in human kidney and brain tumors [29]. MAIT cells have also been proposed to have a protective role during experimental autoimmune encephalomyelitis, a mouse model of multiple sclerosis [17].

Finally, the nature of the antigen(s) recognized by MAIT cells in vivo remains unknown. Evidence suggests that it might not be of peptidic nature, and MAIT cells have been shown to be stimulated by the glycolipid α-mannosylceramide [30]. The significance of these findings remains unclear, as they have been difficult to reproduce [31], and the putative ligand-binding groove of MR1 does not appear especially suited for lipid binding. Nevertheless, the conserved nature of the MAIT cell TCR and its restriction element MR1 suggests the potential recognition of a conserved class of antigenic ligand(s). How this invariant TCR might recognize the antigen/MR1 complex remains unknown. Mutational analysis of MR1 molecules has predicted a traditional diagonal binding of the TCR on MR1 [19]. Similar predictions were made regarding the engagement of the *i* NKT TCR onto CD1d. Yet, the recent crystal structure of a human *i* NKT TCR in complex with α-galactosylceramide presented by CD1d demonstrated a striking engagement of the TCR [32]. It was proposed that the *i* NKT TCR behaves as a pattern recognition receptor and is less “ligand-discriminating” than other TCRs reactive with MHCIa and MHCII molecules [33]. Whether MAIT TCR functions in a similar manner remains to be determined. Such a possibility, given the prominence of these cells in humans, might reveal MAIT cells as a major player in mucosal immune regulation.

## Abbreviations

DN: double negative
DP: double positive
*i*NKT cell: invariant natural killer T cell
MAIT cell: mucosal-associated invariant T cell
MHCI: major histocompatibility complex class I
MR1: MHC-related molecule 1
NKT cell: natural killer T cell
SAP: SLAM-associated protein
SLAM: signaling lymphocytic-activation molecule
TCR: T cell receptor
